# Combination of ^131^I-trastuzumab and lanatoside C enhanced therapeutic efficacy in HER2 positive tumor model

**DOI:** 10.1038/s41598-021-92460-0

**Published:** 2021-06-18

**Authors:** Nagarajan Vinod, Jae Hyung Kim, Seungbum Choi, Ilhan Lim

**Affiliations:** 1 Department of Nuclear Medicine, Korea Cancer Center Hospital, Korea Institute of Radiological and Medical Sciences, (KIRAMS), Seoul, 01812 Republic of Korea; 2 Division of RI-Convergence Research, Korea Institute of Radiological and Medical Sciences, (KIRAMS), Seoul, 01812 Republic of Korea; 3 Department of Radiological & Medico-Oncological Sciences, University of Science and Technology (UST), Seoul, 01812 Republic of Korea

**Keywords:** Cancer immunotherapy, Drug development, Radiotherapy, Targeted therapies, Cancer

## Abstract

Lanatoside C has a promising anti-tumor activity and is a potential candidate for radiosensitizers. In this study, we have investigated the therapeutic efficacy of the combination of ^131^I-trastuzumab and lanatoside C for inhibition of human epidermal growth factor receptor 2 (HER2) positive tumor progression in NCI-N87 xenograft model. The combination treatment (^131^I-trastuzumab and lanatoside C) showed highest cytotoxicity when compared to non-treated control or trastuzumab alone or ^131^I alone or ^131^I-trastuzumab alone in vitro*.* Biodistribution studies using ^131^I-trastuzumab or combination of ^131^I-trastuzumab and lanatoside C showed tumor uptake in BALB/c nude mice bearing HER2 positive NCI-N87 tumor xenograft model. The higher tumor uptake was observed in ^131^I-trastuzumab (19.40 ± 0.04% ID/g) than in the combination of ^131^I-trastuzumab and lanatoside C (14.02 ± 0.02% ID/g) at 24 h post-injection. Most importantly, an antitumor effect was observed in mice that received the combination of ^131^I-trastuzumab and lanatoside C (*p* = 0.009) when compared to control. In addition, mice received lanatoside C alone (*p* = 0.085) or ^131^I-trastuzumab alone (*p* = 0.160) did not significantly inhibit tumor progression compared with control. Taken together, our data suggest that combination of ^131^I-trastuzumab and lanatoside C might be a potential synergistic treatment for radioimmunotherapy to control the HER2 positive tumor.

## Introduction

Radioimmunotherapy (RIT) represents an attractive approach that combines the advantage of radiation therapy and immunotherapy using monoclonal antibodies (mAbs)^1,2^. Currently, the targeted radiation delivered by mAbs kills explicitly cancer cells or the tumor microenvironment^3^. RIT have been used primarily for the treatment of lymphoma, mostly with radiolabeled mAbs against CD20^4–7^. Moreover, Knox et al.^8^ have shown that RIT is more effective than external beam radiation therapy in animal models. Recent research trends found the treatment of tumors using isotope-releasing beta-emitters such as ^90^Y, ^177^Lu, ^131^I and ^124^I^9–11^. The radioactive isotope is selected considering the physical properties such as path length, emission energy, and half-life^11,12^. This is to establish a therapeutic strategy to effectively reduce the size of tumors^2,11^. Among the many radioisotopes used for RIT, ^131^I has advantages of being easy to use. The 8-day half-life of ^131^I increases the efficiency of the treatment, consistent with the biological half-life of the antibody^12,13^. In addition, the path length of the beta-particle of ^131^I is relatively short and effectively treats small tumors. It is also easy to discharge outside the body. However, RIT has the problem of producing radio-resistance tumors in solid tumors and bone marrow toxicity is a problem^2,14^. Therefore, RIT processing capacity is limited and needs to be improved for these problems. Recently several studies have tried to improve therapeutic efficacy of RIT through the use of radiosensitizers^14,15^.

Radiosensitizers are agents that sensitize the tumor cells to radiation^15^. Many drugs and chemicals have been reported as radiosensitizers. Recently, it has been reported that lanatoside C can be used as a radiosensitizer in radiotherapy^16^. Previous studies have shown the effect of lanatoside C as a radiosensitizer at radiotherapy, but its effects with RIT in HER2 positive tumor is not yet known. Therefore, we hypothesis that lanatoside C has an effect of radiosensitizer at ^131^I-trastuzumab RIT in HER2 positive tumor.

In the present study, we investigated the effect of cell proliferation of lanatoside C on two cancer cells (NCI-N87 and MDA-MB231). In addition, the cytotoxicity and therapeutic effects of combined treatment with ^131^I-trastuzumab and lanatoside C were evaluated in HER2 positive cancer cells in vitro and in vivo. 

## Results

### Effect of lanatoside C on cell proliferation of cancer cells

**Figure 1:**
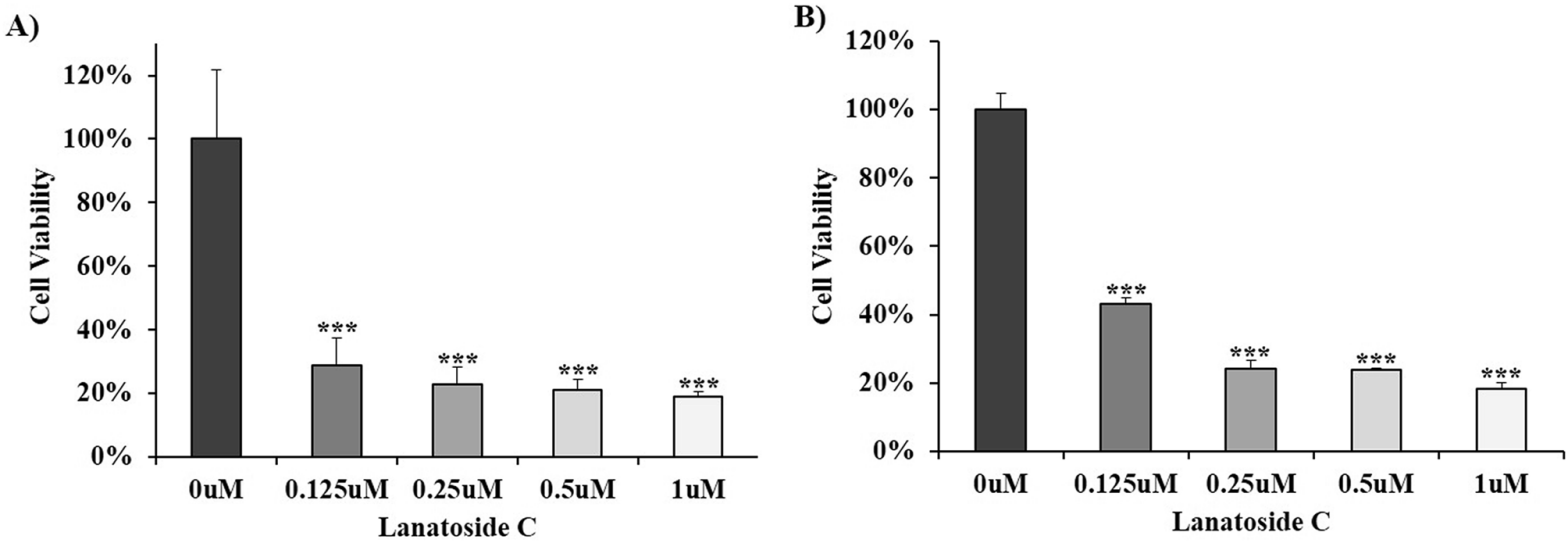
Lanatoside C suppressed growth of cancel cell lines. Inhibitory effect of lanatoside C on cell viability of NCI-N87 (**A**) and MDA-MB231 (**B**) cells. Data are presented as percentage of cell viability in which the 0.01% DMSO treated control sample is set 100%. The average of experimental triplicates ± standard deviation is shown. ***p < 0.001.

Before investigating the ^131^I-trastuzumab in combination with lanatoside C, we determined the cytotoxic effects of lanatoside C in NCI-N87 (HER2 positive) and MDA-MB231 (HER2 negative) cancer cells. Both the cells were treated with various concentrations of lanatoside C and assesses for cell viability using Ez-Cytox cell viability assay. All the doses of lanatoside C shows strong decreases of cell proliferation in both cancer cells when compared to non-treated control cells (*p* < 0.001), suggesting efficient cellular uptake of those lanatoside C concentrations (Fig. 1). Significant decrease of cell viability relative to untreated control was apparent, and most evident following treatment of NCI-N87 with 0.125 nM/well lanatoside C. A notable difference in cell viability was observed between 0.125 µM and 1 µM of lanatoside C. However, no significant difference was found between 0.25 µM and 0.5 µM in both cancer cells.

### Lanatoside C increases the sensitivity of NCI-N87 cells to ^131^I-trastuzumab radioimmunotherapy ***in-vitro***

The cytotoxic effects of lanatoside C on treatment of ^131^I-trastuzumab in NCI-N87 cells was determined using the Ez-Cytox cell viability assay following 96 h incubation.

**Figure 2:**
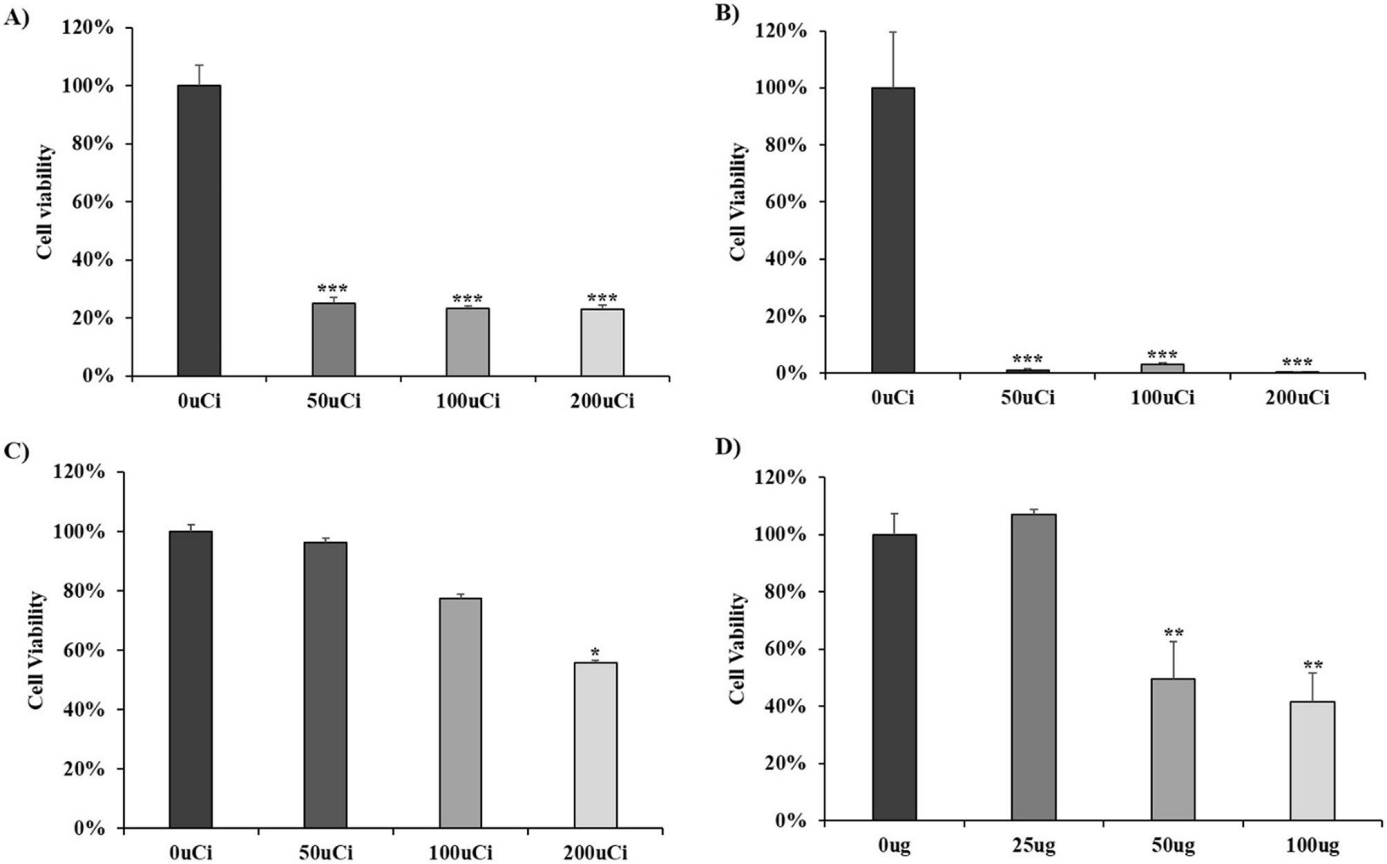
Lanatoside C enhanced ^131^I-trastuzumab RIT in vitro. NCI-N87 cells were treated with various activity of ^131^I-trastuzumab RIT (**A**), ^131^I-trastuzumab RIT combined with lanatoside C (**B**), ^131^I alone (**C**) and trastuzumab alone (**D**), and cell viability was determined by Ez-Cytox assay. Data are presented as percentage of cell viability in which the 0.01% DMSO treated control sample is set 100%. The average of experimental triplicates ± standard deviation is shown. ***p < 0.001, **p < 0.01, and *p < 0.05.

NCI-N87 cells were treated with lanatoside C in combination of ^131^I-trastuzumab RIT. The maximum cell death was found in NCI-N87 cells treated with combination of ^131^I-trastuzumab RIT and lanatoside C (~ 99%) compared to ^131^I-trastuzumab RIT alone (~ 77%) or ^131^I alone (~ 44%) or trastuzumab alone (~ 58%) (Fig. 2). However, no significant differences were observed in the case of 50, 100, 200 µCi of ^131^I-trastuzumab RIT in combination of lanatoside C.

### Lanatoside C increases the sensitivity of NCI-N87 xenografts to ^131^I-trastuzumab radioimmunotherapy ***in-vivo***

**Figure 3:**
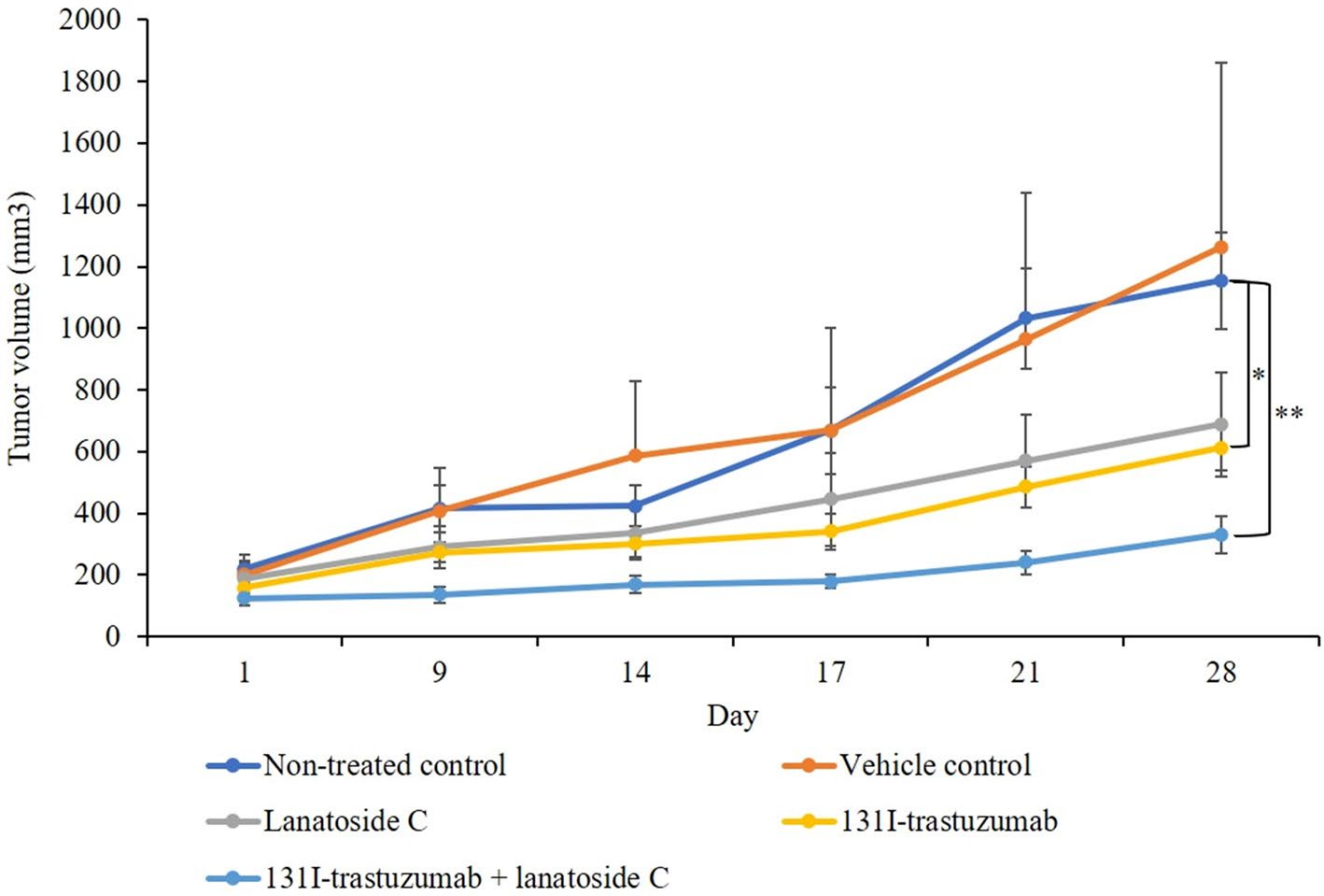
Lanatoside C enhanced ^131^I-trastuzumab RIT in xenograft model. When the NCI-N87 tumor volume reached approximately 150 mm^3^, the BALB/c nude mice were treated with non-treated control group (untreated control), vehicle control group (0.01% DMSO in saline, 100 μl, the first 3 days), lanatoside C (6 mg/kg body weight, the first 3 days intraperitoneal injection), ^131^I-trastuzumab group (400 μCi, once tail vein injection), and combination of ^131^I-trastuzumab and lanatoside C (lanatoside C, 6 mg/kg body weight, the first 3 days intraperitoneal injection, and ^131^I-trastuzumab, 400 μCi, once tail vein injection). **p < 0.01, and *p < 0.05.

The maximum dose of ^131^I for therapeutic efficacy is well known through several studies^17^. Therefore, we performed experiments at a concentration of 400 μCi based on the results of previous studies. We observed that tumor growth rapidly occurred in the vehicle control group administered 0.01% DMSO in saline. Most importantly, it was confirmed that the growth of the tumor was significantly decreased in the group treated with combination of ^131^I-trastuzumab and lanatoside C (*p* < 0.01) and ^131^I-trastuzumab alone (*p* < 0.05) compared to the non-treated control group (Fig. 3). Moreover, there is no significant differences between lanatoside C treated group and the non-treated control group.

### Biodistribution of ^131^I-trastuzumab or combination of ^131^I-trastuzumab and lanatoside C in vivo

**Figure 4:**
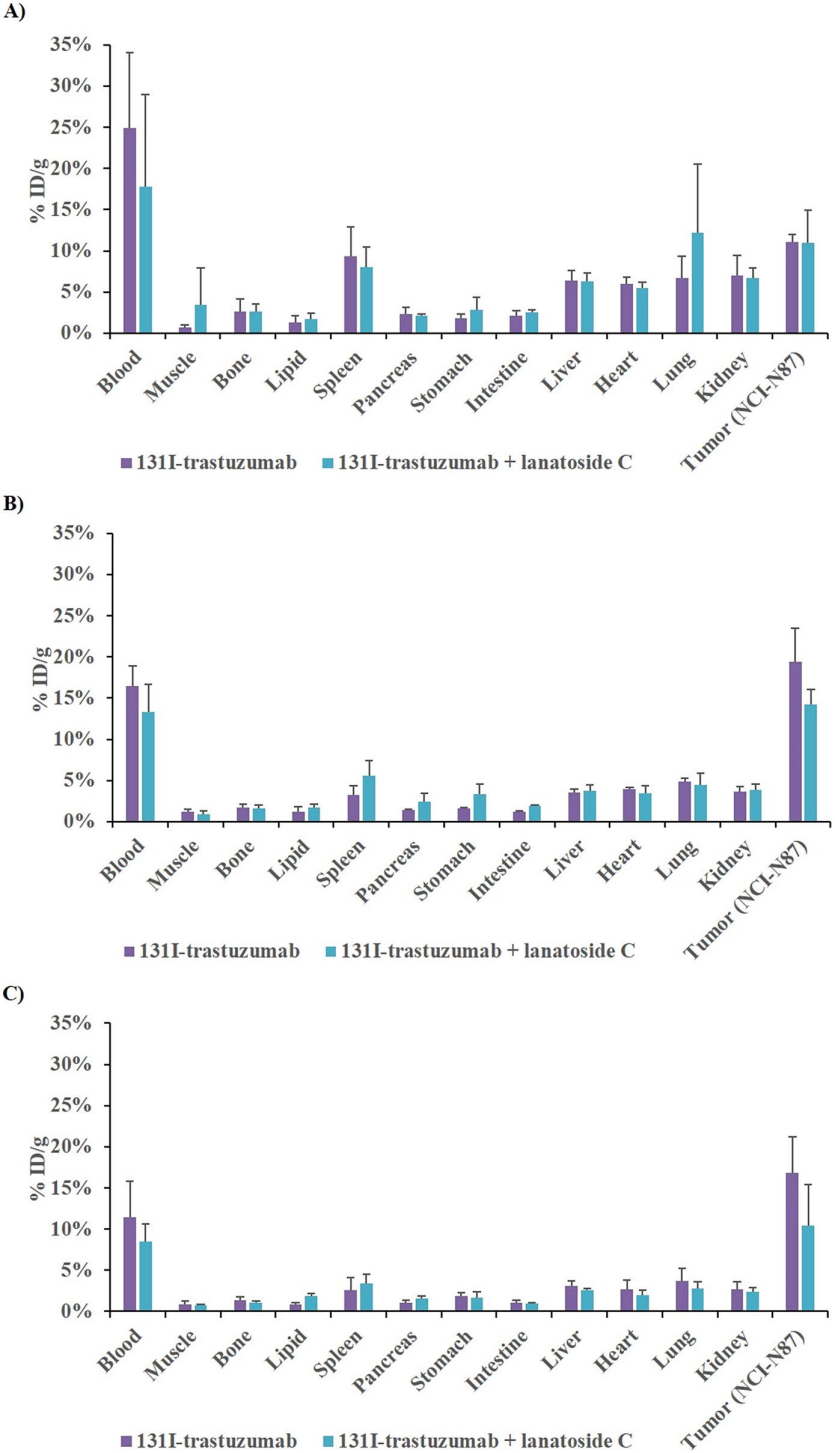
Biodistribution pattern (% ID/g) of ^131^I-trastuzumab or combination of ^131^I-trastuzumab and lanatoside C in tumor (tumor xenograft NCI-N87) bearing BALB/c nude mice at 4 h (**A**), 24 h (**B**), and 48 h (**C**).

The results of the biodistribution data of ^131^I-trastuzumab or combination of ^131^I-trastuzumab and lanatoside C in NCI-N87 xenografted BALB/c nude mice obtained at 4, 24, 48 h post injection (h.p.i) are shown in Fig. 4. Higher uptake of ^131^I was clearly observed in blood, spleen, liver,heart, lung, kidney, and NCI-N87 tumor in ^131^I-trastuzumab alone and combination of ^131^I-trastuzumab and lanatoside C groups at 4 h (Fig. 4A). Notably, ^131^I uptake in the lung was slightly higher in combination of ^131^I-trastuzumab and lanatoside C group (12.14 ± 8.39% ID/g) compared to ^131^I-trastuzumab group (6.70 ± 2.62% ID/g) at 4 h. However, there were no significant differences between groups. The high accumulation of ^131^I in ^131^I-trastuzumab is consistent with extraction of the activity from the blood at 4 h (24.9 ± 0.09% ID/g), 24 h (16.5 ± 0.02% ID/g), and 48 h (11.4 ± 0.04% ID/g). Moreover, the data reveal that NCI-N87 tumor uptake was shown 4 h.p.i in ^131^I-trastuzumab (11.1 ± 0.01% ID/g) with a steady increase through 24 h (19.4 ± 0.04% ID/g) and 48 h (16.8 ± 0.04% ID/g) (Fig. 4A–C). However, combination of ^131^I-trastuzumab and lanatoside C uptake in NCI-N87 tumors at 4 h (10.9 ± 4.04% ID/g), 24 h (14.2 ± 0.02% ID/g) and 48 h (10.4 ± 0.05% ID/g) showed less uptake in NCI-N87 tumors compared with ^131^I-trastuzumab solely at 24 h and 48 h (Fig. 4B,C). However, combination of ^131^I-trastuzumab and lanatoside C group showed significantly higher inhibition of tumor growth compared to ^131^I-trastuzumab alone group (Fig. 3).

## Discussion

The present study demonstrated that the combination of ^131^I-trastuzumab RIT and lanatoside C can improve the therapeutic effects in HER2 positive tumor. We demonstrated that lanatoside C increases the sensitivity of NCI-N87 cells and xenograft models to ^131^I-trastuzumab RIT in vitro and in vivo (Figs. 2 and 3). The results of the present study are consistent with those of earlier studies, in which treatment with lanatoside C led to dose-dependent cytostatic or cytotoxic responses of radiosensitization in two colorectal cancer cell line^16^. Previous studies have shown the effect of lanatoside C as a radiosensitizer at external radiotherapy. However, no study has been conducted in case of RIT. We demonstrated the effect of lanatoside C as a radiosensitizer of ^131^I-trastuzumab RIT in HER2 positive cells (Fig. 2). According to literature, tumor cells are most radiosensitive in the M and G2 phases^18^. Moreover, lanatoside C induces cell cycle arrest in the G2/M phase could be responsible for the difference in radiosensitization^19^. Another study also shown that lanatoside C increased cell sensitivity to radiation by inhibiting DNA damage repair^16^.

Lanatoside C is known to inhibit cell proliferation and induces cell apoposis in tumor cells involving various cellular signaling pathways^19–21^. Additional killing of NCI-N87 cells by ^131^I-trastuzumab could be due to associated high energy beta radiation (0.2 MeV of ^131^I). The enhanced magnitudes of damage are due to the localization of radioisotope very close to cellular targets at membrane and cytoplasm level. The present study also showed that the higher level of cell death measured by Ez-Cytox cell viability, proliferation, and cytotoxicity assay kit was observed after treatment of lanatoside C at various concentrations. Moreover, lanatoside C enhanced ^131^I-trastuzumab RIT in vitro. Combination of ^131^I-trastuzumab and lanatoside C showed highest tumor cell growth inhibition when compared to other groups such as ^131^I-trastuzumab RIT alone, trastuzumab alone and ^131^I alone in NCI-N87 cells. The combinatorial treatment of lanatoside C with ^131^I -trastuzumab RIT would result in higher tumor cell growth inhibition and thus, there is an increase in the number of cell death.

Radionuclide ^131^I emits both β-emission and γ-emission which could be used for radiotherapy. In this work, BALB/c nude mice bearing NCI-N87 tumors were intravenously injected with 400 μCi ^131^I-trastuzumab. It was found that ^131^I-trastuzumab after intravenous injection exhibited obvious tumor accumulation. Moreover, the biodistribution of ^131^I-trastuzumab in mice bearing HER2 positive tumors showed maximum higher tumor uptake at 24 h, but there is no significant difference from combination of ^131^I-trastuzumab and lanatoside C. Biodistribution studies in nude mice showed that ^131^I-trastuzumab targeted the tumors overexpressing the Human HER2 receptor in vivo. ^131^I-trastuzumab accumulated to a significant extent in tumors with % ID/g of 19.4 ± 0.04 in the tumor tissues at 24 h.p.i. which decreased to 16.8 ± 0.045 at 48 h.p.i. Combination of ^131^I-trastuzumab and lanatoside C also showed similar pattern of tumor uptake in mice bearing HER2 positive tumors. Although the combination of ^131^I-trastuzumab and lanatoside C group showed less tumor uptake than the ^131^I-trastuzumab alone group at 24 h.p.i and 48 h.p.i, the combination of ^131^I-trastuzumab and lanatoside C group revealed better treatment response than the ^131^I-trastuzumab alone group. This result might imply that lanatoside C can be considered as a radiosensitizer (Fig. 3). Moreover, there is no significant difference between ^131^I-trastuzumab alone and combination of ^131^I-trastuzumab and lanatoside C at all the time points (4 h, 24 h and 48 h). Steady blood clearance of ^131^I-trastuzumab and ^131^I-trastuzumab combined with lanatoside C demonstrated the stability of the complex under in vivo conditions. The high uptake of ^131^I-trastuzumab by the liver, lungs and spleen may be due to the rich blood flow and its effective metabolism in the reticuloendothelial system of these organs. The tumor uptake and biodistribution ratio of ^131^I-trastuzumab was found to be almost same when compared to combination of ^131^I-trastuzumab and lanatoside C at 4, 24, and 48 h.p.i. However, lung and muscle showed slightly higher uptake in combination of ^131^I-trastuzumab and lanatoside C than ^131^I-trastuzumab at 4 h.p.i. Most importantly, those tissues and other non-targeted tissuses did not show significant differences of uptake between ^131^I-trastuzumab alone and ^131^I-trastuzumab combined with lanatoside C at 4, 24, and 48 h.p.i. The expectation of high tumor uptake of the radiolabeled ^131^I-trastuzumab in HER2 positive tumors was confirmed by the present biodistribution study. Most importantly, combination of ^131^I-trastuzumab and lanatoside C showed better therapeutic effect than ^131^I-trastuzumab or lanatoside C alone in this HER2 positive tumor model. However, there is a limitation because we performed our study only in one HER2 positive tumor model and this might warrant further diverse experiments in other HER2 positive tumor models.

We have performed the clinical trial of RIT for NHL patients using ^131^I-rituximab for 17 years^22–26^. RIT demonstrated excellent outcomes, but there are some refractory patients who revealed resistance to RIT. We expected that it is necessary to treat these refractory patients using more enhanced RIT protocols. We hope that ^131^I-rituximab with lanatoside C can be applied for refractory NHL patients, because we found out that addition of lanatoside C can enhance the RIT of ^131^I-trastuzumab.

## Conclusions

In conclusion, our findings suggest that lanatoside C has the potential to sensitize ^131^I-trastuzumab induced cytotoxicity in NCI-N87 cells in vitro and enhanced strong antitumor effect in a HER2 positive xenograft model. As the results, the combined therapy with ^131^I-trastuzumab and lanatoside C achieved excellent synergistic in vivo therapeutic effects in HER2 positive tumor bearing mice. Therefore, our in vitro and in vivo results provide potentially important and promising therapeutic strategies for future clinical translations in radioimmunotherapy.

## Materials and methods

### Cells and reagents

NCI-N87 and MDA-MB231 cell lines were purchased from the American Type Culture Collection (New York, USA). All these cells were cultured in RPMI-1640 (WELGENE Inc., Daegu, Korea) supplemented with 10% heat-inactivated fetal bovine serum (FBS; Omega Scientific, Inc., Tarzana, CA, USA), 2 mmol/L L-glutamine, 5% Penicillin/Streptomycin in a humidified atmosphere of 5% CO_2_ at 37° C. Lanatoside C was purchased from Sigma-Aldrich (St. Louis, MO, USA). Herceptin (Trastuzumab), A therapeutic agent that targets HER2 (Human Epidermal growth factor Receptor 2) was purchased from Roche. ^131^I was purchased in New Korea Industrial Co., Ltd. Ez-Cytox cell viability, proliferation, and cytotoxicity assay kit was purchased from DoGenBio (Seoul, Korea).

### Radiolabeling

Radiolabeling of trastuzumab with ^131^I was achieved using the Pierce Pre-coated Iodination Tubes (Thermo scientific, U.S.A.) and carried out in accordance with the protocol provided by Thermo scientific^27^. Briefly, the pierce pre-coated iodination tube was wetted with 1 ml of Tris iodination buffer and decanted. 500 µCi of ^131^I was added to the Pierce pre-coated iodination tube and activated for 5 min at room temperature. Subsequently, 100 µg of trastuzumab was added to the tubes and the reaction mixture was incubated for 10 min at room temperature. Radiolabeling purity was determined by instant thin-layer chromatography (Agilent Technologies) using saline. Incorporation purity was always exceeded 95%.

### Determination of the effect of lanatoside C on cancer cells

For the in vitro cell viability assay was carried out according to protocol described by Ez-Cytox cell viability, proliferation, and cytotoxicity assay kit^28^. Briefly, 100 µl of NCI-N87 or MDA-MB231 cells were firstly seeded into 96-well plate for 24 h and then incubated with various concentration of lanatoside C (0.125, 0.250, 0.500 and 1.000 µM). After 96 h incubation, 10 µl of Ez-Cytox solution were added into each well and incubated at 37° C for 0.5–4 h. The absorbance was quantified at 450 nm using a SpectraMax i3 microplate reader (Molecular Devices, Sunnyvale, CA). In this study, MDA-MB231 cells were used for HER2 negative and NCI-N87 cells were used for HER2 positive in this experiment. Therefore, we mainly focused on NCI-N87 cells for further experiments. All experiments were repeated three times with at least triplicate readings for each concentration. Percent cell viability was calculated as the percentage of the ratio of optical density (OD) of treated and 0.01% DMSO treated control samples. Viability (%) = (absorbance of treated sample − blank) / (absorbance of 0.01% DMSO treated control sample − blank) × 100. 

### Estimation of cell death in NCI-N87 cells treated with ^131^I-trastuzumab and lanatoside C in vitro study

Cells were seeded at a density of 2 × 10^4^ cells per well in a 96-well plate and incubated for 24 h at 37° C. After incubation, cells were treated with trastuzumab (0, 25, 50, or 100 µg) or ^131^I alone (0, 50, 100, or 200 µCi) or ^131^I-trastuzumab alone (0, 50, 100, or 200 µCi) or ^131^I-trastuzumab combined with lanatoside C (0, 50 µCi and 0.2 µM, 100 µCi and 0.2 µM, or 200 µCi and 0.2 µM). Control group was treated with 0.01% DMSO. After 96 h of incubation, cell viability was determined using the Ez-Cytox (cell viability, proliferation and cytotoxicity assay kit) following the manufacturer’s instructions. The absorbance was measured at 450 nm using a SpectraMax i3 microplate reader (Molecular Devices, Sunnyvale, CA). All experiments were repeated three times with at least triplicate readings for each concentration. Percent cell viability was calculated as the percentage of the ratio of optical density (OD) of treated and 0.01% DMSO treated control samples. Viability (%) = (absorbance of treated sample − blank) / (absorbance of 0.01% DMSO treated control sample − blank) × 100. 

### Experimental animals

Pathogen-free BALB/c nude mice were obtained from Dooyeol Biotech, Korea. All animal experiments were approved by the Committee for the Handling and Use of Animals and performed in accordance with institutional guidelines at Korea Institute of Radiological and Medical Sciences in compliance with the ARRIVE guidelines.

### Xenograft model

To make a xenograft mouse model, NCI-N87 cells (5 × 10^6^/mouse/0.1 ml) were injected subcutaneously into the dorsal right flank of 6-week-old BALB/c nude mice. When the tumor volume reached approximately 150 mm^3^, the mice were randomly assigned to four groups (7 mice/group): (1) non-treated control group (untreated control: NCI-N87 tumor), (2) vehicle control group (0.01% DMSO in saline, 100 μl, the first 3 days), (3) lanatoside C (6 mg/kg body weight, the first 3 days intraperitoneal injection), (4) ^131^I-trastuzumab group (400 μCi, once tail vein injection), and (5) ^131^I-trastuzumab and lanatoside C combination (lanatoside C, 6 mg/kg body weight, the first 3 days intraperitoneal injection, and ^131^I-trastuzumab, 400 μCi, once tail vein injection). Tumor size and body weight were measured once a week, and the tumor volume (V) was calculated using the following formula: V = L × W^2^/2 (L, long diameter of the tumor; W, short diameter of the tumor).

### Biodistribution Study

The biodistribution of the ^131^I radiolabeled trastuzumab was assessed in BALB/c nude mice bearing established NCI-N87 xenografts. Mice were injected with ^131^I-trastuzumab (400 μCi) or combination of ^131^I-trastuzumab and lanatoside C by tail vein injection. At 4, 24 and 48 h after post-injection (h.p.i), groups of 4 mice were euthanized by isofluorane anesthesia and then immediately bled via cardiac puncture. Tumors and normal tissues (muscle, bone, lipid, spleen, pancreas, intestine, liver, heart, lung, kidney and tail) were then resected and placed in individual γ-counter tubes. The activity of all samples were then counted on a gamma counter (2480 Wizard^2^, PerkinElmer, Waltham, MA, USA), and the percent injected dose per gram (% ID/g) calculated. Results were expressed as Mean ± SD for each time point.

### Statistical analysis

All data are expressed as the mean ± SD and are representative of at least triplicate experiments. The significance was determined using student t test and one way Anova test by using SPSS statistical program. A value of *p* < 0.05 was considered to be significant.

## References

[1] Bethge WA, Sandmaier BM (2005). Targeted cancer therapy using radiolabeled monoclonal antibodies. Technol. Cancer Res. Treat..

[2] Larson SM, Carrasquillo JA, Cheung NK, Press OW (2015). Radioimmunotherapy of human tumours. Nat. Rev. Cancer.

[3] Navarro-Teulon I, Lozza C, Pelegrin A, Vives E, Pouget JP (2013). General overview of radioimmunotherapy of solid tumors. Immunotherapy.

[4] Deshayes E (2013). Tandem myeloablative ^131^I-rituximab radioimmunotherapy and high-dose chemotherapy in refractory/relapsed non-Hodgkin lymphoma patients. Immunotherapy.

[5] Frost SH (2015). Comparative efficacy of 177Lu and 90Y for anti-CD20 pretargeted radioimmunotherapy in murine lymphoma xenograft models. PLoS ONE.

[6] Hohloch K (2014). Radioimmunotherapy for first-line and relapse treatment of aggressive B-cell non-Hodgkin lymphoma: An analysis of 215 patients registered in the international RIT-Network. Eur. J. Nucl. Med. Mol. Imaging.

[7] Reagan PM, Friedberg JW (2015). Advancing radioimmunotherapy and its future role in non-Hodgkin lymphoma. Fut. Oncol..

[8] Knox SJ, Goris ML, Wessels BW (1992). Overview of animal studies comparing radioimmunotherapy with dose equivalent external beam irradiation. Radiother. Oncol..

[9] Kairemo KJ (1996). Radioimmunotherapy of solid cancers: A review. Acta Oncol..

[10] Kraeber-Bodere, F. *et al.* in *Seminars in Oncology.* 613–622 (Elsevier).

[11] Sharkey RM, Goldenberg DM (2011). Cancer radioimmunotherapy. Immunotherapy.

[12] Kelly MP (2009). Therapeutic efficacy of 177Lu-CHX-A"-DTPA-hu3S193 radioimmunotherapy in prostate cancer is enhanced by EGFR inhibition or docetaxel chemotherapy. Prostate.

[13] Luster, M., Pfestroff, A., H채nscheid, H. & Verburg, F. A. in *Seminars in Nuclear Medicine.* 126–134 (Elsevier).

[14] Kumar S, Singh RK, Meena R (2016). Emerging targets for radioprotection and radiosensitization in radiotherapy. Tumor Biol..

[15] Ghotra VP, Geldof AA, Danen EH (2013). Targeted radiosensitization in prostate cancer. Curr. Pharm. Des..

[16] Kang MA (2016). Lanatoside C suppressed colorectal cancer cell growth by inducing mitochondrial dysfunction and increased radiation sensitivity by impairing DNA damage repair. Oncotarget.

[17] Klein JL (1989). Yttrium-90 and iodine-131 radioimmunoglobulin therapy of an experimental human hepatoma. Cancer Res..

[18] Pawlik TM, Keyomarsi K (2004). Role of cell cycle in mediating sensitivity to radiotherapy. Int. J. Radiat. Oncol. Biol. Phys..

[19] Reddy D, Kumavath R, Ghosh P, Barh D (2019). Lanatoside C induces G2/M cell cycle arrest and suppresses cancer cell growth by attenuating MAPK, Wnt, JAK-STAT, and PI3K/AKT/mTOR signaling pathways. Biomolecules.

[20] Hu Y (2018). Lanatoside C inhibits cell proliferation and induces apoptosis through attenuating Wnt/beta-catenin/c-Myc signaling pathway in human gastric cancer cell. Biochem. Pharmacol..

[21] Durmaz I (2016). Liver cancer cells are sensitive to Lanatoside C induced cell death independent of their PTEN status. Phytomedicine.

[22] Kang GW (2013). Radioimmunotherapy with (131)i-rituximab in a patient with diffuse large B-cell lymphoma relapsed after treatment with (90)y-ibritumomab tiuxetan. Nucl. Med. Mol. Imaging.

[23] Kang HJ (2011). Radioimmunotherapy with (131)I-rituximab for patients with relapsed/refractory B-cell non-Hodgkin's lymphoma (NHL). Asia Pac. J. Clin. Oncol..

[24] Lee I (2019). Comparisons of (131)I-rituximab treatment responses in patients with aggressive lymphoma and indolent lymphoma. Ann. Nucl. Med..

[25] Lim I (2013). Prognostic significance of pretreatment (1)(8)F-FDG PET/CT in patients with relapsed/refractory B-cell non-Hodgkin's lymphoma treated by radioimmunotherapy using (1)(3)(1)I-rituximab. Acta Haematol..

[26] Shin DY (2016). Radioimmunotherapy with (131)I-rituximab as consolidation therapy for patients with diffuse large B-cell lymphoma. Cancer Chemother. Pharmacol..

[27] Lee YS, Kim JS, Cho KD, Kang JH, Lim SM (2015). Tumor dosimetry for I-131 trastuzumab therapy in a Her2+ NCI N87 xenograft mouse model using the Siemens SYMBIA E gamma camera with a pinhole collimator. J. Instrum..

[28] Kang M, Jeong CW, Ku JH, Kwak C, Kim HH (2014). Inhibition of autophagy potentiates atorvastatin-induced apoptotic cell death in human bladder cancer cells in vitro. Int. J. Mol. Sci..

